# Epidemiological, Genomic and In Vitro Insights into the Clinical Significance of *Yersinia enterocolitica* Biotype 1A

**DOI:** 10.3390/pathogens15101015

**Published:** 2026-09-25

**Authors:** Lucia Rivas, Bridget Armstrong, Aruni Premaratne, Jan Powell, Craig Billington, Jing Wang, Jacqueline (Jackie) Wright, Ernest Williams, Brent Gilpin

**Affiliations:** 1 New Zealand Institute for Public Health and Forensic Science (PHF Science), Christchurch Science Centre, 27 Creyke Road, Ilam, Christchurch 8041, New Zealandaruni.premaratne@phfscience.nz (A.P.); jan.powell@phfscience.nz (J.P.); brent.gilpin@phfscience.nz (B.G.); 2 New Zealand Institute for Public Health and Forensic Science (PHF Science), Kenepuru Science Centre, 34 Kenepuru Drive, Kenepuru, Wellington 5022, New Zealand; jing.wang@phfscience.nz; 3 New Zealand Institute for Public Health and Forensic Science (PHF Science), Wallaceville Science Centre, 66 Ward Street, Wallaceville, Upper Hutt 5018, New Zealand

**Keywords:** yersinia, epidemiology, cell adhesion, virulence, biotype 1A

## Abstract

Yersiniosis is a notifiable disease in Aotearoa|New Zealand, which includes *Yersinia enterocolitica* (YE) Biotype (BT) 1A, whose pathogenicity and clinical significance has been questioned. An analysis of yersiniosis cases with different YE biotypes (BTs) notified between 2018 and 2022 identified that YE BT 1A and YE BT 2/3 were more frequently observed among adults of European or Other ethnicity and young children of Asian ethnicity, respectively. A comparison of virulence-associated genes detected using whole-genome sequencing data from YE isolates confirmed that YE BT 1A lacked several recognized virulence genes. In vitro two-dimensional intestinal (Caco-2) cell assays showed that YE BT 1A (n = 4) from clinical cases attached and invaded at significantly lower levels than YE BT 2/3 and 4 (n = 3). Gut three-dimensional organ-on-a-chip infections performed using single isolates of YE BTs 2/3, 5, and 1A showed that the YE BT 1A isolate elicited a significantly larger interleukin-8 inflammatory response than the YE BT 2/3 or 5 isolates, which may suggest an acute inflammatory-mediated intestinal disease mechanism. While further work is required to elucidate the role of YE BT 1A in causing gastrointestinal illness, these findings highlight the need to distinguish cases infected with YE BT 1A from those infected with other YE BTs.

## 1. Introduction

Yersiniosis is a gastrointestinal illness caused by the bacteria *Yersinia enterocolitica* (YE), and less frequently, *Y. pseudotuberculosis* (YP). Yersiniosis can cause diarrhea, vomiting, fever, and abdominal pain. Sepsis may occur in immunocompromised individuals [1]. Other uncommon clinical manifestations and sequalae of yersiniosis include necrotizing enterocolitis in infants, reactive arthritis and erythema nodosum [2].

*Y. enterocolitica* can be characterized as one of six internationally recognized biotypes (BTs) (1A, 1B, 2, 3, 4, and 5) [3]. The key virulence factors associated with the pathogenicity of YE are well described in the literature and include a virulence plasmid (pYV) and a chromosomal attachment and invasion locus (*ail*), amongst other relevant genes [4,5,6,7]. In contrast, YE Biotype (BT) 1A most often lacks the recognized YE virulence genes [8,9]. Several studies have used cell-line and animal assays to elucidate the role of selected virulence genes for the attachment and invasion of *Yersinia* spp. in causing gastrointestinal disease [10,11,12,13,14,15,16], but the question of the pathogenicity of YE BT 1A continues to be unclear and controversial [8,9]. Recent technological advancements, including whole-genome sequence (WGS) analysis, can infer several different virulence profiles and, in combination with three-dimensional (3D) microphysiological assays (such as organ-on-a-chip assay), can be used to better assess host–pathogen interactions between isolates of interest [17].

Unlike many countries, in Aotearoa|New Zealand (New Zealand), yersiniosis is a notifiable disease under the Health Act 1956 and includes YE BT 1A detections. A case–control study and WGS assessment conducted in 2021–2022 reported an association and genomic links with raw pork and YE BT 2/3 (serotype O:9 and correlating to multi-locus sequence type (ST) 12) that represented 45% of cases. In contrast, the 27% of cases that had YE BT 1A were not associated with pork, but rather with other unconfirmed food sources, and clinical case isolates were genomically very diverse (46 different multi-locus sequence types among 85 isolates), with only four pairs of clinical isolates showing close genomic relationships. This was despite the high frequency (>50%) of YE BT 1A isolated from foods including raw meat, and with almost two-thirds of food samples with pathogenic YE BTs found to also contain YE BT 1A, as well [18].

In addition, a follow-up questionnaire of yersiniosis cases within the case–control study identified epidemiological trends among those notified with YE BT 1A and other BTs, including more adults notified with YE BT 1A and children notified with YE BT 2/3. Cases of YE BT 1A also tended to self-report more pre-existing gastrointestinal conditions, and/or had a greater uncertainty or inability to identify a start date of when symptoms associated with yersiniosis began compared with those notified with YE BT 2/3 [19]. Collectively, these observations from New Zealand studies add to the speculation from other international studies that question the true association of YE BT 1A with gastrointestinal symptoms or whether it is an incidental finding in clinical cases [20,21], as well as studies examining whether YE BT 1A, or a subset, may be associated with gastrointestinal disease, especially in immuno-compromised individuals, with alternative virulence mechanisms causing different symptom outcomes [2,9,10,22,23].

We report the first use of an organ-on-a-chip ‘gut’ assay, alongside a traditional two-dimensional (2D) cell-line assay to examine the ability of different YE BTs to attach and invade the human Caco-2 cell line. An assessment of the virulence profiles among YE BTs and an assessment into epidemiological trends across cases notified during 2018–2022 were also performed. The objective of the study was to explore evidence supporting the pathogenic potential of YE BT 1A and therefore its continued public surveillance.

## 2. Materials and Methods

### 2.1. Yersiniosis Notification Data Analysis

A notified case in New Zealand represents a person with clinical illness who has presented at a healthcare facility and provided a stool sample that has tested positive for yersiniosis. A confirmed case of yersiniosis in New Zealand is defined as a clinically compatible illness accompanied by laboratory definitive evidence of either (i) isolation of YE or YP from blood or feces or (ii) detection of *Yersinia* spp. nucleic acid from feces [1]. Case report form data from confirmed yersiniosis cases notified from 1 January 2008 to 31 December 2022 was extracted from the EpiSurv database 14 June 2024. The data was analyzed for annual incidence, and the following quantitative and qualitative variables, along with the Statistics New Zealand mid-year general population estimates, were included: case age, sex, ethnicity, and health district based on census data collected for 2018 and 2023 [24,25]. A sub-population analysis according to ethnicity and age group was also performed. Where population estimates for the sub-population were not available for non-census years, linear interpolation was used to estimate sub-population counts for those years. The average annual incidence rate was calculated over the 2018–2022 timeframe. Ethnicity numbers were based on prioritized classification of self-determined ethnicity grouped in order of Indigenous Māori, Pacific People, Asian, Middle Eastern/Latin American/African (MELAA), and New Zealand European [26]. For this study, those identified as European (including New Zealander) and MELAA were grouped into ‘European or Other’. Case epidemiology was also assessed by regions, which were defined by Health New Zealand|Te Whatu Ora to include: (1) Northern|Te Tai Tokerau, (2) Midland|Te Manawa Taki, (3) Central|Te Ikaroa, and (4) South Island|Te Waipounamu.

Whether the date of onset of illness was recorded or not for yersiniosis cases notified between 2018 and 2022 was analyzed and compared with the date of notification of yersiniosis in EpiSurv. As the time required for collection, transport, and testing of stool samples and the subsequent notification into EpiSurv typically requires at least 24 h, cases that had the same date recorded for onset of illness and notification were assigned ‘no’ for date of illness reported.

### 2.2. Virulence Gene Detection Using Whole-Genome Sequencing Data

All YE isolates from notified yersiniosis cases associated with a New Zealand case–control study underwent whole-genome sequence analysis as previously described [18] and were used for virulence gene detection. In brief, all isolates were grown on Tryptic Soya Agar (TSA; Fort Richard, Auckland, New Zealand) at 28 °C for 24 h ± 2 h. Bacterial growth was collected from the plate and used to inoculate 1 mL Tris-EDTA buffer to a density of 2–4 McFarland Standard, which was then heat inactivated at 65 °C for 1 h. These suspensions were stored at 4 °C until genomic DNA was extracted using the Chemagic^™^ 360 extraction platform (PerkinElmer, Waltham, MA, USA). DNA quality and concentration analysis was performed using PicoGreen (Quant-iT; Thermo Fisher Scientific, Waltham, MA, USA). Sequencing libraries containing 1 ng of DNA were prepared using Nextera XT chemistry (Illumina, San Diego, CA, USA) for 150 bp pair-end sequencing on an Illumina NextSeq sequencer, according to the manufacture’s recommendations (Illumina).

Raw sequence reads for all isolates analyzed in this study are available at the National Centre for Biotechnology Information (NCBI) short read archive (SRA) with BioProject number PRJNA 1142067. The genomes of two YE BT 5 isolates from sick animals, as previously described [27], were also included in the analysis.

Initial sequence quality and species identification using the Illumina data were determined using the Nullarbor version 2 pipeline [28]. The ST was inferred using WGS data via the McNally scheme for YE and Achtman scheme for YP [29,30]. Isolates to be included in the current study were selected to cover a range of different *Yersinia* species and YE BTs and seven-gene multi-locus sequence types (STs) as previously observed [18]. Virulence-associated genes were inferred using the WGS data via ABRIcate and the Virulence Factor Database (VFDB) (version 1.01) [31,32,33], using a minimum DNA percentage identity and coverage of 80% and 60%, respectively. Assembled draft genomes were also annotated using Bakta (version 1.10.1), and an in-house Python (version 3.9) script was used to extract and analyze the *ail* gene [34]. The presence of plasmids was determined for isolates used in the cell-assays only (see Section 2.3) using MOB-Suite (version 3.1.6) [35].

### 2.3. In Vitro Intestinal Cell Assays

#### 2.3.1. Bacterial Cultures

Eight YE isolates were selected for the 2D Caco-2 adhesion and invasion assays, of which three were subsequently selected for further assessment using the 3D organ-on-a-chip assay. For the 2D Caco-2 assay, a single isolate of clinical origin was selected for each of the following YE bioserotypes: YE BT 2/3, O:9 (ST12; 16ER3959) and O:5,27 (ST14; 22ER3657) and YE BT 4, O:3 (ST18; 22ER1765). These bioserotypes possessed the key virulence genes (namely ail and genes associated with the pYV and TTSS) recognized for cell attachment, invasion, and pathogenesis. For the 3D organ-on-a chip assay, the YE BT 2/3 O:9 (ST12; 16ER3959) was selected for testing as it represented the most common ST causing yersiniosis in New Zealand.

For YE BT 1A, four isolates (ST291,22ER1202; ST192, 22ER3398; ST754, 22ER3870; ST278, 22ER3685) from clinical cases were selected for screening using the 2D Caco-2 adhesion and invasion assay based on the observation that these isolates clustered closely with at least one other clinical isolate of the same ST [18]. The four YE BT 1A isolates demonstrated similar adhesion and invasion abilities within the 2D assay. YE BT 1A (ST192, 22ER3398) was selected for further assessment using the 3D organ-on-a-chip assay as it was observed to possess the same virulence gene profiles as the other YE BT 1A isolates tested in the 2D assay, but also possessed additional genes (*galE*, *gmd*, *rfbF*, *rfbG*, *cpsG*) associated with either lipopolysaccharide/capsule or O-antigen biosynthesis.

A single YE BT 5 isolate (ST13, 18ER1119), originally from a sick animal, was selected for both the 2D cell assay and 3D organ-on-chip assay, as it represented a YE BT that is very rarely observed to cause human infection, yet possesses the key virulence genes (namely *ail* and genes associated with the pYV and TTSS) recognized for cell attachment, invasion, and pathogenesis [27]. It was of interest to observe the behavior of this YE BT in both assay types.

Each YE isolate was grown in tryptic soya broth (TSB; Fort Richard Laboratories, Auckland, New Zealand) at 28 °C with shaking (150 rpm) for approximately 18 h (±2 h). An aliquot of 0.2 mL overnight culture was used to inoculate 7 mL TSB and incubated at 28 °C with shaking (200 rpm) until an optical density (OD) of 0.2 (600 nm) was reached (~1 × 10^8^ colony forming units (CFU)/mL). For the YE BT 5 isolate, it was observed to have a slower growth rate in these conditions; therefore, three sub-cultures were prepared as above and were pooled prior to centrifugation for 10 min at 5000× *g* at 4 °C, and the pellet was resuspending in TSB to achieve OD 0.2. Each culture was centrifuged for 10 min at 5000× *g* at 4 °C, and the supernatant was removed before the pellet was washed once by adding 10 mL phosphate-buffered saline (PBS, Thermo Fisher Scientific), centrifuged, and the supernatant removed again. The final pellet was resuspended in 7 mL Dulbecco’s Modified Eagle Medium (DMEM) (Thermo Fisher Scientific). The initial inoculum levels were determined by serially diluting (1:10) each prepared inoculum into PBS and subsequently spread-plating 100 µL of each dilution onto TSA. All plates were incubated at 37 °C for 18–48 h prior to enumerating colonies.

#### 2.3.2. Two-Dimensional Adhesion and Invasion Assay

Colorectal cancer Caco-2 cells were obtained from the PHF Science cell culture collection and seeded in DMEM, supplemented with heat-treated 20% (*v*/*v*) fetal bovine serum (Thermo Fisher Scientific) and 1% (*v*/*v*) Penicillin–Streptomycin (10,000 U/mL) solution (Sigma-Aldrich, Lab Supply, Dunedin, New Zealand) in T25 tissue culture flasks (Thermo Fisher Scientific) in a humidified 37 °C incubator with 5% CO_2_. The media was changed every two days until the cell monolayer reached 80% confluence. Cells were washed with PBS and then incubated with 3 mL of 0.05% Trypsin EDTA (Thermo Fisher Scientific) for 5 min, resuspended in 7 mL complete DMEM, centrifuged at 200× *g* for 5 min, and the pellet resuspended in complete DMEM. Caco-2 cells were seeded into two six-well collagen-I-coated culture plates (Thermo Fisher Scientific; 3 mL per well, 1 x 10^5^ cells/mL) incubated at 37 °C with 5% CO_2_ until the cell monolayer reached 90–100% confluence. Medium from each well was removed and washed three times using 1 mL PBS.

Adhesion and invasion assays were performed as previously described [13,36], with some modifications. One mL of prepared inoculum for each isolate was inoculated into the six-well plate in duplicate at an estimated multiplicity of infection (MOI) of 100. One six-well plate was used for the adhesion assay, and the other plate was used for the invasion assay. A control consisted of medium without YE inoculum. Plates containing the YE inoculum were incubated at 37 °C with 5% CO_2_ for 2 h to allow for adhesion of the YE to the Caco-2 cells. The medium in each well was aliquoted into a 1.5 mL tube and used to analyze the presence of cytokines (see below). Each well was washed three times with 1 mL PBS to remove any non-adherent bacteria.

One of the two six-well plates was used to determine the number of bacteria that adhered to Caco-2 cells. For each well, 1 mL 0.1% (*v*/*v*) Triton X-100 in PBS (Sigma-Aldrich) was added, and the plates were incubated at room temperature for 5 min to lyse the Caco-2 cells. The cell lysates were mixed by pipetting up and down 10 times, and the lysate was subsequently serially diluted (1:10) in PBS. A 100 µL aliquot of each dilution was spread-plated onto TSA, incubated at 37 °C for 24–48 h, and colonies enumerated to obtain the number (CFU) of adherent bacteria per mL.

The other plate was used to determine the number of bacteria that invaded the Caco-2 cells. For each well, 1 mL DMEM containing 200 µg/mL gentamicin was added and incubated at 37 °C with 5% CO_2_ for 1 h to eliminate extracellular bacteria. Each well was washed three times with 2 mL of PBS, and the PBS was removed. Caco-2 cells were lysed with 1 mL 0.1% (*v*/*v*) Triton X-100 in PBS and colonies were enumerated using the same method as above to obtain the number (CFU) of intracellular bacteria per mL. Each YE isolate was tested in duplicate wells (technical replicates) in each plate, and the experiment was repeated at least three independent times (biological replicates). The percentage of adhesion and invasion was calculated for each isolate as follows:

% adhesion = (CFU/mL adherent bacteria/CFU/mL initial inoculum) × 100.

% invasion = (CFU/mL intracellular bacteria/CFU/mL initial inoculum) × 100.

### 2.4. Three-Dimensional Organ-on-a-Chip Assay

#### 2.4.1. Preparation and Seeding of Organ-on-a-Chip

An Emulate Chip-S1^®^ Stretchable Chip (Emulate, Boston, MA, USA) was used for the experiment as it represents a relevant gut model that mimics peristalsis, improves cell differentiation, and increases tightness of the epithelial barrier compared with 2D or transwell assays. The chips were prepared for cell seeding according to manufacturers’ instructions (Emulate protocol for Caco-2 intestine-chip culture protocol; EP 208 v2.0). Briefly, chips were placed in the Emulate-supplied cradle in a 12 cm^2^ sterile dish (GR688161, MediRay New Zealand, Auckland, New Zealand), and the top and bottom channels of the chips were filled with ER-1 solution (Emulate) and then exposed to ultra-violet (UV) light (Emulate; model NS-01-US) for 10 min to activate the chips. The ER-1 solution was removed from the channels, which were refilled with fresh ER-1 solution and exposed to UV for an additional 5 min. Both top and bottom channels were washed with ER-2 buffer (Emulate) and then PBS. The activated channels were coated with extra cellular matrix (ECM), prepared by mixing Matrigel (Corning^®^ 354234, Bio-Strategy, Auckland, New Zealand) and collagen Type 1 (Corning^®^ 354249, Bio-Strategy) in PBS buffer. ECM-coated chips were incubated at 37 °C and 5% CO_2_ overnight.

Human umbilical vein endothelial cells (HUVEC; PCS-100-013, In Vitro Technologies, Auckland, New Zealand) representing the vascular cell line were grown and passaged in vascular cell basal medium (PCS-100-030, In Vitro Technologies) supplemented with endothelial cell growth kit VEGF (PCS-100-041, In Vitro Technologies) to 80% confluence, washed with PBS, and then incubated with 3 mL of 0.05% Trypsin EDTA (Thermo Fisher Scientific) for 3 min. Cells were resuspended in 7 mL vascular cell media, centrifuged at 200× *g* for 5 min, and the pellet resuspended in the minimum volume of complete vascular cell growth media to achieve a live cell count of 6.0 × 10^6^ cells/mL. Twenty µL of the HUVEC suspension was seeded into the bottom channel of the chip, and 50 µL of complete DMEM media without cells added into the top channel of the chip. The chip was inverted and incubated at 37 °C, 5% CO_2_ for 2 h. Caco-2 cells were prepared as for HUVEC but resuspended in complete DMEM to achieve a live cell count of 3 × 10^6^ cells/mL. A volume of 50 µL Caco-2 cell suspension was seeded into the top channel of the ECM-coated chip and incubated for 2 h. A gravity wash was performed as recommended within the manufacturer’s instructions and chips were incubated at 37 °C, 5% CO_2_, 18 h. Chips were subsequently attached to the Emulate Pod^®^ with a Emulate Zoë^®^ Culture Module and a regular cycle for overnight with a media flow rate of 30 µL/h was applied without stretching. On day 3, 3 mL each of complete DMEM and vascular cell media were replenished in the top and bottom inlet reservoirs, respectively, and 10% chip stretch at 0.15 Hz was added. Media reservoirs were refilled every second day until Caco-2 cells formed tight junctions (~6 days), which was confirmed by the Cascade blue barrier function assay described by the Emulate protocol EP187_v1.0. Barrier function was tested before and after the infection by collecting 50 µL of media from each reservoir, which was transferred to a 96-well plate in triplicate, and fluorescence intensity was measured using a spectrophotometer (CLARIOstar; BMG LABTECH GmbH, Ortenberg, Germany; excitation: 390/15 nm, emission 440/20 nm).

#### 2.4.2. Organ-Chip Infection

Each experiment had duplicate organ-chips for each isolate (technical replicates) and negative control (DMEM only without inoculum), for a total of eight chips, and was performed twice on separate days (biological replicates). Three YE isolates (16ER3959, BT 2/3; 18ER1119, BT 5; and 22ER3398, BT 1A) were each grown in 20 mL TSB, at 28 °C for 18 h, and 200 µL was used to sub-culture 7 mL TSB, which was grown at 28 °C until log-phase (OD_600_ 0.2; equivalent 1.0 × 10^8^ CFU/mL). A seeded organ-chip was removed from the Emulate Pod^®^, and the top channel was washed once with 200 µL DMEM without antibiotics, while the bottom channel was washed with 200 µL vascular cell medium without antibiotics. The top channel was inoculated with 100 µL of a single YE culture, which provided an estimated MOI of 100 based on the number of Caco-2 cells seeded into the chip, while 100 µL vascular cell medium was added to the bottom channel. All inoculated organ-chips were incubated at 37 °C with 5% CO_2_ for 2 h under static conditions. Subsequently, the top channel for each organ-chip was washed once with 200 µL DMEM, followed by 100 µL DMEM containing 200 µg/mL gentamicin and further incubation for 2 h. The top channel was subsequently washed twice with 200 µL DMEM. The organ-chips were returned into the Emulate Pod^®^ containing DMEM with 10 µg/mL of gentamicin in both top and bottom channel reservoirs and run for 24 h under the regular cycle (30 µL/h media, 10% stretch, 0.15 Hz). Following the cycle, for each chip, 100 µL of effluent from each of the top and bottom channels (including control) were spread-plated onto TSA, incubated at 37 °C for 48 h, and subsequently examined for colonies. The remaining effluent in each of the top and bottom channels was collected and analyzed using quantitative PCR (qPCR) for YE and cytokine presence (see below).

#### 2.4.3. Quantitative PCR (qPCR) for YE

To ascertain whether either YE invaded and crossed from the top (Caco-2 cells) to the bottom (HUVEC) channel following incubation, effluents from both channels were collected separately, and YE was detected and semi-quantified using qPCR. Each effluent was centrifuged at 5000× *g* for 10 min, and DNA was extracted from the bacterial pellet using the DNeasy Blood & Tissue Kit (Qiagen, Venlo, The Netherlands) on a QIAcube (Qiagen) according to the manufacturer’s instructions. Each DNA extraction was screened by qPCR using the gene target *foxA* [37] in a LightCycler 480 II system (Roche, Auckland, New Zealand) with SYBR green qPCR Master Mix (Roche). A standard curve was generated from serial dilutions of gBlocks (Integrated DNA technologies, Singapore) of the gene to enable quantification.

### 2.5. Cytokine Presence

The presence testing and semi-quantification of interleukin (IL)–6, IL-8, IL-10, and tumor necrosis factor-alpha (TNF-α) were performed using matched antibody pairs and protein standards (BD Biosciences, Franklin Lakes, NJ, USA) within a sandwich enzyme-linked immunosorbent assays (ELISA) according to manufacturer’s instructions. The media collected during the 2D Caco-2 adhesion and invasion experiments and 3D organ-chip experiments were centrifuged at 10,000× *g* for 10 min at 4 °C to obtain cell-free supernatants. For the 2D experiments, cell-free supernatants were obtained for 16ER3959, BT 2/3 (n = 4); 22ER1765, BT 4 (n = 6); four BT1A: 22ER3685 (n = 4), 22ER3398 (n = 8), 22ER3870 (n = 8), and 22ER1202 (n = 2); and the control (n = 2) (after the 2 h incubation used for the adhesion step only), while supernatants for 18ER1119, BT 5 and 22ER3657, BT 2/3 were not available for cytokine testing. The amount of IL expressed (pg/mL) was calculated in comparison with a standard curve generated with a protein standard contained in the kit.

### 2.6. Statistical Analysis

Statistical analyses of data, including linear interpolation, were performed within R v4.3 software using Tidyverse packages [38,39]. Epidemiological data was assessed for significance where appropriate using either chi square or Fisher’s exact test. For the 2D and 3D cell-assay experiments, including cytokine presence, all datasets were assessed for normality using the Shapiro–Wilk test, and the homogeneity of variances was assessed using Levene’s test. Due to several datasets exhibiting non-normal distributions, unequal variances, small sample sizes, or zero-valued observations, non-parametric statistical methods were applied throughout. For comparisons involving more than two groups (e.g., individual isolates and/or with controls, or YE BTs), differences were assessed using Kruskal–Wallis rank-sum test, which represents the non-parametric equivalent of a one-way analysis of variance and does not require the assumption of normally distributed data. Where the Kruskal–Wallis test indicated a significant overall effect, pairwise comparisons were performed using Dunn’s post hoc test with Holm-adjusted *p*-values. Statistical significance was accepted at *p* < 0.05.

For the 2D and 3D cell experiments, including cytokine presence, Microsoft 365 Copilot (CPT-5-based model, Microsoft Corporation) was used to assist with statistical workflow planning, generation of R code for data visualization and statistical analyses, and some interpretation of statistical outputs. All statistical analyses and figures were independently executed and verified by the authors using R. AI tools were not used for study design, data collection, or generation of research findings and discussion.

## 3. Results

### 3.1. Analysis of Notified Cases of Yersiniosis

Figure 1 provides an overview of the number of human yersiniosis cases between 2008 and 2022. An increase in the proportion of notified cases identified as YE BT 2/3 and BT 1A and a decrease in the proportion of notified cases identified as YE BT 4 have been observed since 2013. The proportion of notified yersiniosis cases without BT information increased from 11% (138/1199) of cases in 2018 to 32% (380/1185) of cases in 2019 and remained within the range of 23–29% for all cases across 2020–2022. Ninety-nine percent (2590/2595) of cases without BT information were due to the inability to isolate YE or YP from a PCR-positive case sample, and therefore no isolate was sent to the Enteric Reference Laboratory (ERL) for typing. Six isolates were confirmed as YE, but a BT was not able to be identified. Seven case isolates were identified as *Y. hibernica* (n = 2), *Y. fredicksenii* (n = 1), or *Y. fenwicki* (n = 4), which do not meet the case definition for yersiniosis and were excluded from further analyses. There were eight YE BT 1B (O:8) reported in this timeframe but also excluded from further analyses due to low numbers. International travel history was not provided with the dataset. However, it was observed that the yersiniosis notifications were not impacted by the border and travel restrictions that occurred as a result of the COVID-19 pandemic [40].

The average annual incidence rate (referred to as rate(s) hereafter) of yersiniosis for New Zealand between 2018 and 2022 was 25.3/100,000 (total cases 6345). The rate for cases notified in 2018–2022 did not differ between females (25.2/100,000) and males (25.3/100,000). A similar rate for YE BT 2/3 was observed for females (11.8/100,000) and males (12.2/100,000). However, the rate for YE BT 1A was higher for females (4.3/100,000) compared with males (3.5/100,000), while the opposite was observed for YE BT 4 (males: 3.5/100,000, females: 2.4/100,000). The rates for YE BTs 2/3 and 4 between 2018 and 2022 were highest for children under five years of age (47.4/100,000 and 19.0/100,000, respectively) compared to any other age-group (ranging 6.7/100,000–12.8/100,000 for YE BT 2/3 and 1.1–4.2/100,000 for YE BT 4).

In New Zealand, ethnicity is a self-identified classification. In 2018, the New Zealand population was 4,699,755, of which 68% were reported as European, 18% Māori, 17% Asian, 9% Pacific People, and 3% other [24]. The rate of yersiniosis between 2018 and 2022 was highest for cases that identified as Asian (33.4/100,000), followed by European or Other (21.5/100,000), and Māori and Pacific People represented the two ethnic groups with the lowest rates (14.2/100,000 and 12.6/100,000, respectively).

Rates for YE BT 2/3 and 4 stratified according to age-group and Asian or European or Other ethnicity are shown in Figure 2. With the exception of adults over 70 years of age, the rates for YE BT 2/3 were consistently higher for cases that identified as Asian across all age-groups (ranging from 10.5/100,000 to 88.4/100,000) compared with those that identified as European or Other (ranging from 5.5/100,000 to 28.5/100.000). For children under five years of age, YE BTs 2/3 and 4 rates were approximately three and twelve times higher, respectively, among Asian children (88.4/100,000 and 45.7/100,00, respectively) than those identifying as European or Other (28.5/100,000 and 3.7/100,000, respectively).

Of the 861,000 New Zealand residents identifying as Asian in 2018, the two major ethnicities were Chinese (32%) and Indian (34%) [24]. It was not possible to calculate rates for these Asian sub-populations; however, between 2018 and 2022, a total of 1091 cases notified with YE BTs 2/3 and 4 and identified as Asian also provided further ethnicity information. Of these, 58% (n = 634) identified as Chinese only, while 7% (n = 77) identified as Indian only.

For YE BT 1A, the rates were highest in cases over 70 years of age (7.3/100,000), with rates progressively increasing for each age-group from age 10–15 years to 60–69 years old (1.3/100,000–5.4/100,000). The rate for YE BT 1A was highest for those cases that identified as European or Other for age-groups over 20 years of age (3.9/100,000–7.2/100,000), compared with those that identified as Asian in the same age-groups (1.8/100,000–4.8/100,000) (Figure 2).

Both Māori and Pacific People had higher rates for YE BT 2/3 (6.5/100,000 and 6.0/100,000, respectively) compared with other YE BTs (1A or 4). Children under the age of five years old that identified as Māori had the highest rates for YE BT 2/3 (23.8/100,000 and 10.9/100,000, respectively) compared with other age-groups within the same ethnicity (ranging from 2.3/100,000 to 8.7/100,000). For Pacific People, cases within 60–69 years of age had the highest rate for YE BT 2/3 (12.6/100,000), followed by children under the age of five (10.9/100,000), whilst the rates among the age-groups ranged from 1.3/100,000 to 8.4/100,000.

New Zealand is divided into four health regions: Northern|Te Tai Tokerau, Midland|Te Manawa Taki, Central|Te Ika Roa, and the South Island|Te Waipounamu, comprising approximately 38%, 20%, 19%, and 23% of the New Zealand population, respectively (2022/2023) [41]. Figure 3 outlines the rates for different YE BTs (and cases without typing information) notified during 2018–2022 according to health region. The highest rate for YE BT 1A was observed in the South Island|Waipounamu region (7.1/100,000) compared with other health regions (2.6/100,000–3.9/100,000). In contrast, the highest rate for YE BT 2/3 was observed in the Northern|Te Tai Tokerau region (13.0/100,000) compared to other regions (10.6/100,000–11.9/100,000). The rate for YE BT 4 was lowest in South Island|Te Waipounamu (1.3/100,000) compared with other regions (2.8/100,000–3.7/100,000). The rate for cases without BT information was highest for the Midland|Te Manawa Taki and Central|Te Ika Roa regions (9.5/100,000 and 9.8/100,000, respectively) compared with other regions (3.9/100,000–4.1/100,000).

A date for the onset of illness was recorded for 48% (3069/6345) of yersiniosis cases notified between 2018 and 2022 (Table 1). Among the YE BTs (1A, 2/3, 4), there was a small but statistically significant (*p* = 0.03: chi-square test) difference in the proportion of cases with a recorded onset date, although the differences between YE BTs were modest (42–47%). The median number of days between onset date and the notification did not vary between the YE BTs (11–13 days). The proportion of cases with onset-to-notification delay of 30 or more days differed between YE BT (*p* < 0.0001; chi-square test), with higher proportions observed among cases notified with YE BT 1A (21%) and those without BT identified (19%), compared with YE BT 2/3 (6%) and YE BT 4 (3%). Cases that were notified with YP had a higher completion rate of onset date (50/68; 74%) compared with any YE BT (42–46%), with approximately 10% of cases (5/50) reporting ≥30 days between date of onset of illness and notification.

Whether a case was hospitalized for yersiniosis was recorded for 63% (4036/6347) of yersiniosis cases between 2018 and 2022 (Table 1). There was no statistical difference (*p* = 0.2; chi-square test) in the proportion of cases notified with YE BT (1A, 2/3, or 4) and reported hospitalization, ranging between 13 and 15%. Hospitalization associated with YP was a small sample size (n = 64), but pairwise comparisons using Fisher’s exact test indicated a significantly (*p* < 0.0001) high proportion of YP cases hospitalized (33%) compared with any YE BT.

### 3.2. Virulence Gene Analysis from Whole Genome Sequencing Data

Virulence gene analysis was performed on a total of 470 YE genomes that were predominately obtained from a New Zealand case–control study. Table 2 outlines the key YE virulence-associated genes that differ across the YE BTs in the dataset analyzed in this study. The presence or absence of these key virulence-associated genes detected were similar for clinical and food isolates for each of the YE BTs. The *ail* output using the VFDB was negative for all YE genomes, but positive for YP genomes. Further annotation and extraction of the *ail* gene from all genomes identified that 100% of YE BT 2/3 (n = 167), YE BT 4 (n = 15), and the two YE BT 5 animal genomes possessed the *ail* gene, while the YE BT 5 genomes contained a 27 bp insertion within the *ail* gene. All YE BT 1A (n = 286) genomes were confirmed negative for the *ail* gene using the annotation approach. Using the VFDB, several genes associated with plasmid pYV, including those within the Type III secretion system (TTSS), were identified in 100% of YE BTs 2/3, 4, and 5 genomes, while all were absent in all YE BT 1A genomes.

*InvA* was detected in 99–100% of YE genomes. In contrast, *InvB* was detected only in YE BT 1A, and then only in 82% (235/286) of genomes. The *invB* was partially detected in one of the two YE BT 5 genomes. The *ystA* enterotoxin gene was present in all genomes of YE BT 2/3, 4, and 5, but was absent in all YE BT 1A isolates. In contrast, the *ystB* enterotoxin gene was detected in all YE BT 1A isolates, but was absent in all YE BT 2/3, 4, and 5 genomes. Several capsule/exopolysaccharide or O-antigen biosynthesis genes (*cpsG*, *galE*, *gmd*, *rfbF*, *rfbG*) were observed at variable frequencies (18–31%) amongst the YE BT 1A isolates, while they were absent in all YE BT 2/3, 4, and 5 genomes. The four YE BT 1A isolates used for the cell assays had similar virulence gene profiles, except for ST192, which possessed *cpsG*, *galE*, *gmd*, and *rfbG*, which were absent in the other YE BT 1A isolate. Therefore, the ST192 was selected for subsequent 3D cell assessment. In addition, the YE BT 1A ST278 isolate was lacking the *invB* gene, while the other YB BT 1A possessed the gene.

Additional toxin genes including *yaxA* and *yaxB* were detected in all YE BT 2/3, 4, and 1A genomes. Despite the YE BT 5 genomes presenting with many of the same virulence genes as YE BT 2/3 and 4, the two YE BT 5 genomes did not possess *yaxA*, while *yaxB* was partially present. The *yaxB* and the *yscP* genes were the only virulence genes that differed between the genomes from YE BT 2/3, 4, and 5 isolates used for the cell assays.

The presence of plasmids was assessed for the YE isolates used in the cell assays only. The pYV was confirmed within the genomes of the YE BT 2/3, 4, and 5 isolates used, while no plasmids were detected with the YE BT 1A isolates. The YE BT 5 isolate also had an additional plasmid AD917 detected.

### 3.3. In Vitro 2D Cell Assays

Eight YE isolates representing different BTs and STs were assessed for intestinal cell attachment and invasion using an in vitro 2D Caco-2 cell assay. The initial inoculum (~8.2 log_10_ cfu/mL) used for each isolate (n = 3–5 per isolate) did not significantly differ between YE isolates tested (Kruskal–Wallis test; H = 3.4, *p* = 0.85), indicating that any subsequent differences in adhesion and/or invasion were not attributed to variation in the initial bacterial load. The percent adhesion and invasion of a selection of YE isolates to Caco-2 cells is presented in Figure 4. Some replicates, particularly for YE BT 2/3 ST14, achieved percentage of adhesion values of >100%, which may indicate either high variability in viable counts on plating media or possible growth of YE during the 2 h incubation period, resulting in more adherent bacteria than the initial inoculum. However, the ability or rate of growth for the YE isolates in the medium and experimental conditions used for the cell assays were not independently determined. The levels of adhesion differed significantly between YE isolates (*p* < 0.01; Kruskal–Wallis test; H = 21.5, *p* = 0.003, n = 27). Each isolate representing YE BT 2/3 ST12, BT 2/3 ST14, and BT4 ST18 exhibited substantially greater adhesion percentages than the YE BT 1A (n = 4) isolates and the YE BT 5 isolate. However, statistical significance following Dunn’s post hoc testing with Holm correction was demonstrated for YE BT2/3 ST14 versus BT5 ST13 (*p* = 0.02) and BT4 ST18 versus BT5 ST13 (*p* = 0.03). YE BT2/3 ST12 showed a similar trend but did not reach significance after adjustment (*p* = 0.063). No other pairwise comparisons remained significant following adjustment for multiple testing. Invasion differed significantly among YE isolates (Kruskal–Wallis test: H = 19.3, *p* = 0.007; n = 27 ). However, no pairwise differences remained significant following Dunn’s multiple-comparison test with Holm adjustment (all adjusted *p* > 0.05).

A significance difference in adhesion and invasion was observed when observations were grouped according to YE BT (BT 1A, n = 14; BT 2/3, n = 6; BT 4, n = 4; BT 5, n = 3; total n = 27 ), as determined by a Kruskal–Wallis test (adhesion: H = 20.7, *p* = 0.0001 and invasion: H = 18.7, *p* = 0.0003). Post hoc pairwise comparisons using Dunn’s test with Holm adjustment demonstrated significantly greater adhesion for YE BT 2/3 isolates compared with YE BT 1A (adjusted *p* = 0.006), YE BT 4 compared with YE BT 1A (adjusted *p* = 0.04), YE BT 2/3 compared with YE BT 5 (adjusted *p* = 0.001), and YE BT 4 compared with YE BT 5 (adjusted *p* = 0.006). No significant differences were detected between YE BT 2/3 and YE BT 4 (adjusted *p* = 0.833) or between YE BT 1A and YE BT 5 (adjusted *p* = 0.179). Using the same test, significantly greater invasion was observed for YE BT 2/3 compared with YE BT 1A (adjusted *p* = 0.012) and YE BT 5 (adjusted *p* = 0.027). Similarly, YE BT 4 exhibited significantly greater invasion than YE BT 1A (adjusted *p* = 0.007) and YE BT 5 (adjusted *p* = 0.015). No significant differences were detected between YE BT 2/3 and YE BT 4 (adjusted *p* = 1.0) or between YE BT 1A and YE BT 5 (adjusted *p* = 0.58). The YE BT 5 isolate was also observed to have very low adhesion (0.1%) and invasion (0%), comparable to the YE BT 1A isolates (ranging from 1.3 to 11.8% for adhesion and 0.4–0.1% for invasion).

Cell-free supernatants available for six YE isolates from selected 2D assays were collected after the adhesion period (2 h) and were assessed for an inflammatory response to YE. Of the inflammatory cytokines tested, IL-8 was the only one detected within the 2D cell assay supernatants using the ELISA method (IL-6, IL-10, and TNF-α were not detected). IL-8 concentrations differed significantly among YE isolates and the control (*p* < 0.01; Kruskal–Wallis; n = 34). However, no individual isolate differed significantly from the control after correction (all adjusted *p* > 0.05; Dunn’s test with Holm adjustment). Although isolates YE BT 2/3, YE BT 1A ST192, and YE BT 1A ST754 exhibited higher IL-8 concentrations than the control, the small number of control replicates limited statistical power (adjusted *p* > 0.05; post hoc Dunn Test with Holm adjustments). A comparison of IL-8 concentrations between YE isolates only (control excluded) indicated a significant difference between YE BT 2/3 and YE BT 1A ST278 (adjusted *p* = 0.004; post hoc Dunn Test with Holm adjustments), YE BT 1A ST192 and YE BT 4 (adjusted *p* = 0.008), and YE BT 2/3 and YE BT 4 (adjusted *p* < 0.001) (Figure 5).

Analysis of the four YE BT 1A isolates only showed significant differences in IL-8 concentrations (Kruskal–Wallis test: H = 13.7, *p* = 0.003; n = 22). Dunn’s post hoc testing with Holm adjustment demonstrated that YE BT 1A ST192 had significantly greater IL-8 concentrations than YE BT 1A ST278 (adjusted *p* = 0.004), while YE BT 1A ST754 also induced significantly greater IL-8 concentrations than YE BT 1A ST278 (adjusted *p* = 0.04). No other pairwise comparisons were statistically significant.

### 3.4. In Vitro 3D Organ-on-a-Chip

Three selected YE isolates representing YE BTs 2/3, 1A, and 5 were assessed using differentiated Caco-2 /HUVEC chips. Following 24 h of infection, none of the YE isolates caused barrier permeability from the Caco-2 cell (top) channel to the HUVEC (bottom) channel (Figure 6). Although a larger variability in permeability was observed for the control chips (n = 4), the mean permeability in all YE-infected chips (n = 4 for each isolate) was not significantly different between YE isolates or the control (Kruskal–Wallis test: H = 3.66, *p* = 0.3; n = 16). Chips infected with YE isolates had tight barrier function (low permeability), suggesting that none of the YE isolates disrupted the epithelial/endothelial gut barrier following 24 h of infection. In addition, no YE bacterial cells or YE-specific nucleic acid were detected within the effluents collected from the HUVEC (bottom) channel of the chips, indicating that YE did not traverse the epithelial (Caco-2) and endothelial (HUVEC) cell layers. Detection of the YE-gene target in the supernatants collected from the Caco-2 (top) channel confirmed the presence of bacteria associated with the epithelial cell layer .

Based on the inflammatory cytokine response in 2D Caco-2 cells, selected supernatants collected from the Caco-2 (top) channel and HUVEC (bottom) channel from the chips were assessed for human IL-8 by ELISA following completion of the experiments (~24 h) (Figure 7). IL-8 concentrations differed significantly among the isolates in both the Caco-2 (top) channel (Kruskal–Wallis test: H = 22.8; *p* < 0.001) and HUVEC (bottom) channel (Kruskal–Wallis test: H = 22.3; *p* < 0.001). Dunn’s post hoc analysis with Holm-adjusted *p*-values demonstrated that YE BT 1A ST192 showed significantly greater IL-8 concentrations than the control in both the Caco-2 (top) channel (adjusted *p* = 0.0003) and the HUVEC (bottom) channel (adjusted *p* = 0.0002). No significant differences between YE BT 2/3 ST12 or YE BT 5 ST13 and the corresponding control were observed (all adjusted *p* > 0.05). The IL-8 concentrations also differed more significantly for YE BT 1A ST192 than YE BT 5 ST13 for both the Caco-2 (top channel) and HUVEC (bottom) channels (adjusted *p* = 0.0004 and *p* = 0.0006, respectively), while no other isolate-to-isolate comparisons were statistically significant (*p* > 0.05).

## 4. Discussion

A previous New Zealand case–control study of 247 notified yersiniosis cases found that YE BT 1A notifications predominated amongst adults, while YE BT 2/3 notifications were observed more often in children [18]. The current study confirms these epidemiological trends in a much larger dataset of over 6000 yersiniosis cases notified in New Zealand between 2018 and 2022. Moreover, this study identifies ethnic, sex, and health region differences in the rates of yersiniosis among cases with different YE BTs.

For most notifiable enteric diseases in New Zealand, annual incidence rates have historically been lower among individuals identifying as Asian compared with those classified as European or Other [43]. Yersiniosis represents a notable exception to this pattern. For example, in 2022, incidence rates for campylobacteriosis, salmonellosis, and Shiga-toxin-producing *Escherichia coli* infections were 52.7, 13.2, and 8.0 per 100,000 population, respectively, among the Asian ethnic group, compared with 135.6, 16.1, and 25.8 per 100,000 among the European or Other group. In contrast, the incidence of yersiniosis was higher among the Asian population (34.9 per 100,000) than among the European or Other group (23.5 per 100,000) [44].

In this study, the higher rates of yersiniosis among individuals identifying as Asian in New Zealand were attributable to higher incidence of YE BTs 2/3 and 4, rather than YE BT 1A, with disparities most pronounced in younger age groups. Among children under five years of age, up to three times as many YE BT 2/3 and YE BT 4 cases were notified in the Asian population than in the European or Other population, despite the Asian population being approximately one-quarter the size of the European or Other population in New Zealand.

This illness burden was also not evenly distributed across Asian sub-populations. Despite a similar population of Chinese and Indian individuals in New Zealand, cases of YE BT 2/3 were over-represented in those who identified as Chinese, while relatively fewer cases identified as Indian. This finding indicates that there is a substantial and potentially modifiable burden amongst cases of Chinese ethnicity. These patterns are further shaped by geographic clustering, with two-thirds (63%) of New Zealand’s total Asian population in 2018 residing in the Northern|Te Tai Tokerau health region [24], where higher notification rates of yersiniosis, particularly YE BT 2/3, were also observed.

A New Zealand case–control study attributed YE BT 2/3 to exposure to raw pork [18]. Consumption of pork in New Zealand has decreased from 26.3 kg per capita in 2018 to 24.5 kg per capita in 2022 [45]. However, there is limited information as to the amounts and types of pork, including further processed pork products, consumed by different ethnic groups in New Zealand, especially as a significant amount of pork is further processed into cured meats, which potentially has less risk for yersiniosis. Demographic characteristics, including sex and age groups, of the consumption of meat in New Zealand have been previously reported, where both male and older participants had indicated a higher consumption level of pork compared with females and younger participants, respectively [46]. Cultural and religious factors influence the meat choices for Indian migrants to New Zealand, with these groups often limiting pork consumption [47], which correlates to the low yersiniosis incidence rates reported above. In contrast, Chinese consumers in New Zealand have been reported to consume significantly greater quantities of lamb and pork than New Zealand consumers [48]. Further insight is required to elucidate consumer behavior, particularly pork source, consumption and preparation practice, that may explain the higher exposure rate of YE amongst ethnic groups.

The pathogenicity of YE BT 1A has long been an issue of uncertainty; it is often considered as non-pathogenic in many countries [10] yet is notifiable in New Zealand. The reasons to explain the higher notification rate observed for YE BT 1A and adults, particularly females in the South Island|Te Waipounamu region of New Zealand, remain unclear. This study also observed more YE BT 1A cases reported ≥30 days between onset of illness and notification date, compared with other YE BTs as previously reported [19,22]. The hospitalization rates were similar across cases notified with YE BTs 2/3, 4, and 1A. Details of pre-existing gastrointestinal conditions and reasons for hospitalization (including dual infections) were not available within the epidemiological data analyzed in this study, but other studies, including a New Zealand study, have indicated that infections with YE BT 1A are more common in individuals who are immune-suppressed or elderly, suggesting that YE BT 1A may be an opportunistic pathogen that may rely on the state of the host defense system to cause illness [2,23,49]. Outbreaks of YE BT 1A reported in Australian residential old-age facilities were linked to nutritionally supplemented milkshakes, highlighting the foodborne risk of YE BT 1A to a vulnerable population [49].

Several studies have used various cell-line or animal models to assess the pathogenicity of YE, including BT 1A, all with variable results [10,11,12,13,14,15]. The ability of YE to invade epithelial cells is an important correlation to virulence [10]. Overall, all YE BT 1A genomes analyzed in this study lacked several recognized virulence genes associated with the pathogenic YE BTs (2/3 and 4), consistent with other reported findings [5,7,50,51,52,53,54]. In this study, the attachment and invasion of selected YE BT 1A isolates to undifferentiated 2D Caco-2 cells was less than that of YE BTs that are considered pathogenic (2/3 and 4) and concurs with previous reports with HeLa or HEp-2 cell lines [55,56]. Cell attachment and invasion did not differ significantly among the four YE BT 1A isolates despite their distinct sequence types. The four YE BT 1A isolates had similar virulence gene profiles, except for the YE BT 1A ST192, which had additional genes (*galE*, *gmd*, *rfbF*, *rfbG, cpsG*) associated with either lipopolysaccharide/capsule or O-antigen biosynthesis [54,57,58]. A previous study reported that the YE O:3 O-antigen can affect the exposure and function of surface adhesins (particularly *InvA* and *YadA*), indicating that the O-antigen influences host–pathogen interactions through surface architecture, rather than simply increasing invasion [58].

To our knowledge, this is the first study to report the infection of a 3D differentiated Caco-2 /HUVEC organ-on-a-chip model with YE. Observations from these assays showed no evidence of movement of either YE isolate tested (irrespective of BT) from the apical epithelial cell surface (top channel) to the vascular channel (bottom), or disruption of barrier function. This data supports the observation of others that YE is able to invade undifferentiated cells but not differentiated cells [59]. However, in contrast to the 2D cell assay, the 3D organ-on-a-chip assay revealed the ability of the selected YE BT 1A ST192 to elicit an acute IL-8 response from the endothelial/vascular cell (HUVEC) compartment, which was significantly higher than YE BT 2/3 (ST12) or BT 5 (ST13). It is not known whether the release of IL-8 from HUVECs in response to YE BT 1A ST192 was due to direct interaction with bacterial factors (e.g., released toxin/protein) or indirectly by signaling from the epithelial cells. While beyond the scope of the current study, this observation warrants further investigation to determine the potential mechanisms and commonality among a larger selection of YE BT 1A isolates, other YE isolates, and defined genetic mutants. Furthermore, variability in IL-8 results between the 2D and 3D assays may be due to methodological differences, including the incorporation of both epithelial and endothelial cells, and the notable difference in the infection period: 2 h for 2D and 24 h for 3D. Previous studies have shown that YE can invade 2D Caco-2 cells within hours [60], and that IL-8 secretion depends on cell adhesion rather than on bacterial invasion in 2D cervical cell (HeLa) culture [61].

Pro-inflammatory response of YE has been previously reported, and adhesin A (*invA*) and *yadA* contribute to the induction of IL-8 expression and secretion in 2D HeLa cells [62]. The *invA* gene is recognized as the most efficient invasion factor in promoting the tight binding and uptake of bacteria into mouse Payer’s patches [63,64]. Assessment of virulence-associated genes using WGS data indicated that *invA* was present in all YE isolates, irrespective of BT, but *yadA* was absent in all YE BT 1A, while present in other YE BTs. However, an alternative invasin (*invB*, also known as *Ifp*) gene was present in the majority (80%) of YE BT 1A genomes. *InvB* has been described as a novel adhesin in YP that has been reported to have a high homology to *InvA*. Experiments involving a murine coinfection model showed that the colonization of Payer’s patches and the mesenteric lymph nodes of mice by the *ifp*-deficient strain was significantly reduced, and fewer bacteria reached the liver and spleen [65]. The *InvB* gene is expressed at late log phase at 37 °C, suggesting that InvB is a late-stage adhesin [66]. The role of this gene in the attachment or invasion of YE in human cells has not been elucidated. A previous study had indicated that invasin (*inv*-) gene homologues in non-pathogenic strains of YE are non-functional [67]. Acute release of IL-8 in response to some enteric pathogens can lead to a neutrophil-mediated infiltration, epithelial transmigration, and resultant intestinal diarrheal response [68]. Addition of human polymorphonuclear blood cells to the organ-on-a-chip may elucidate whether YE BT 1A can cause a disease phenotype via inflammatory rather than toxigenic mechanisms.

Two genes that encode for YST enterotoxin B and A, *ystB* and *ystA*, respectively [69,70], were notably different between the YE BTs analyzed in this study. All YE BT 1A possessed *ystB*, and not *ystA*, while the contrary was observed for YE BTs 2/3 and 4 and animal isolates of YE BT 5. The widespread distribution of *ystB* in human clinical isolates of YE BT 1A has been previously reported [27,71,72]. The ability of YE BT 1A to produce YstB enterotoxin under ileal conditions (37 °C, pH 7.5) has shown that *ystB* may be an important virulence factor for YE BT 1A [70]. One study reported that the toxicity of YstB was greater than YstA and induced clinical symptoms comparable to those caused by typical YE infections [69,73]. However, there is no current evidence to suggest that severity/occurrence of disease is linked to the presence of *ystB*. The observation of *ystB* in other non-YE species such as *Y. intermedia*, *Y. frederiksenii*, and *Y. kristensenii* has also been reported [74].

The *ail* gene and the pYV are key determinants attributed to the attachment and invasion of YE to cell-lines, yet these determinants are commonly lacking in YE BT 1A [16,27,71,72,75,76]. However, some studies have reported the presence of *ail* in YE BT 1A [75,77,78], with a recent report describing the gene to be located on the genomes of prophages [79]. Interestingly, an isolate of YE BT 5 (a BT that is rarely reported in human cases and historically reported in sick animals [27]) that possessed *ail*, as well as many of the other key virulence genes observed in YE BTs 2/3 and 4, was observed to poorly attach and invade 2D Caco-2 cells. On further genomic analysis, the *ail* gene in the YE BT 5 genome had a 27 bp insertion, which differed from the *ail* gene in YE BT 2/3 and 4 isolates, which was highly conserved. Previous studies have reported different sequence types for the *ail* among different YE BTs [80,81]. It is speculated that the unique sequence of the *ail* gene reflects specialization in this structure important to the invasion process, but adherence by itself is not sufficient to promote entry into the cell [51]. Mutations in the *ail* in YE were also observed to reduce the ability of YE to invade tissue culture cells and significantly reduce their ability to survive the bactericidal effects of serum [53]. It is plausible that *ail* variants influence reception recognition, colonization efficiency, and survival within particular host species (i.e., animal versus human), but further research is required to confirm this hypothesis.

The notable absence of *yaxA* and *yscP* from the YE BT 5 genomes compared with the other pathogenic BTs (2/3, 4) supports evidence that these two genes may be critical for the successful attachment and invasion of YE to human cell lines. However, caution must be taken as only two YE BT 5 genomes were analyzed in this study, and both isolates were of animal origin. The YaxAB complex represents a family of pore-forming toxins, whereby *yaxA* and *yaxB* are both required for the cytotoxin activity of mammalian cells by inducing cell lysis through the formation of pores in the host membrane [82]. A study showed that a strain of YE with the toxin orthologue YaxAB knocked out altered colonization and tissue pathogenic behavior in a murine infection model [82]. Crystal structures of YaxA and YaxB and cryo-electron microscopic mapping of the YaxAB complex have shown a pore structure predominately composed of decamers of YaxA-YaxB heterodimers. Although both subunits were observed to have membrane-active moieties, only YaxA was able to bind to the membranes by itself. YaxB is reportedly recruited to membrane-associated YaxA and induced to present its lytic transmembrane helices and continue with pore formation [83]. Interesting, all YE BT 1A isolates in the current study possessed *yaxA* and were observed to attach and invade more than the YE BT 5 isolate, which was *yaxA*-negative. The precise role of YaxAB in mammalian YE infection remains unknown [83]; it is therefore plausible that this is a key gene associated with pathogenicity in humans. In addition, despite possessing all virulence-associated genes with TTSS, the YE BT 5 genome lacked the *YscP*, a gene that encodes a protein required for the delivery of the Yop effectors, within the TTSS, into macrophages. This system enables the bacteria to weaken the immune defense of the host and allows extracellular-located bacteria that are in close contact to eukaryotic cells to deliver toxic bacterial proteins into the cell [84]. In addition to the pYV, the AD917 plasmid was identified for the YE BT 5 isolate used in the cell assays. This plasmid has been previously described to be associated with sugar fermentation and observed in pork isolates [50].

Virulence gene assessment in this study was based on interrogation of virulence gene databases [32]. Therefore, putative genes absent from these databases, or genes involved in regulating virulence-gene expression, may not have been screened [4,51,81,82]. As observed for *ail*, sequence variation among species [80,81] and the detection thresholds applied may also influence gene identification using VFDB and other bioinformatic databases. It is also critical to highlight that the presence of any virulence gene may not reflect their expression under the conditions used in the cell models [85].

A limitation to the current study was the small number of YE isolates used in the cell assays. Although virulence-gene profiles were similar between clinical and food isolates within each YE BT, only selected clinical isolates were tested. Therefore, it cannot be confirmed whether food isolates would show the same phenotypes in 2D cell and 3D organ-on-a-chip assays. Previous studies have reported differences in virulence and behavior between clinical and environmental strains [14,51]. A New Zealand case–control study did observe certain YE BT 1A STs more frequently in source (food) isolates compared with clinical isolates, and it has been previously suggested that different pathogenic lineages or more human-adapted strains may exist within YE BT 1A [9,86]. The genetic heterogeneity of YE BT 1A is well recognized, with one study indicating that this BT may represent more than one subspecies [9]. Caution is therefore required when extrapolating the results from the selected few YE BT 1A isolates used in the current study. However, findings support the view that the absence of well-recognized virulence genes may limit the ability of YE BT 1A to attach to and invade experimental cell lines. It is plausible that YE BT 1A may use alternative virulence-associated factors, such as *ystB*, to cause disease with symptoms similar to those caused by other YE BTs when host defenses are compromised [10]. The medium infective dose of *Yersinia* spp. in humans is not known, but it is estimated to be 10^4^–10^6^ organisms [87]. The infective dose and clinical presentations of the symptoms may depend on the YE BT and/or strains and host factors. Further assessments would be required to elucidate the role of the gene differences observed between YE BTs, with a greater number of isolates than used in this study, especially as there is a paucity of information with respect to YE BT 5, which may help with deciphering the key virulence genes associated with pathogenicity of other BTs.

The New Zealand case definition for yersiniosis includes isolation of YE or YP from blood or feces. Information on the clinical sample type tested was not provided with the clinical case dataset analyzed in this study. However, overall confirmed laboratory sample information for 2018–2022 indicated that of the 60 YE isolates confirmed from blood samples, none were identified as YE BT 1A, while 54 were YE BT 2/3 or 4, and 6 were YP. However, YE BT 1A was confirmed in a single aspirate sample (, personal communication, Ernest Williams). Previous data showed that <1% of yersiniosis cases (2012–2019) were extra-intestinal, but YP was disproportionally higher than YE amongst those cases [27], indicating that YP is disproportionally notified with more severe disease [88]. At the time of publication, the New Zealand yersiniosis case definition is under review, with a proposal to expand the case definition to include extra-intestinal cases to improve data capture for cases with severe disease.

In a New Zealand study, interviewed yersiniosis cases within the case–control study showed that the number of cases that self-reported a pre-existing gastrointestinal condition(s) was higher for those confirmed with YE BT 1A (49%; 23/47) compared with those with YE BT 2/3 (24%; 21/86) [19]. Of those cases reporting pre-existing condition(s), more cases reported with YE BT 1A (36%: 15/45) than YE BT 2/3 (12%; 10/86) indicated conditions that included IBS, colitis, ulcerative colitis, Crohn’s disease, coeliac, and bowel conditions . Information on pre-existing gastrointestinal condition(s) of notified cases is not routinely available within the surveillance process in New Zealand and was a limitation within the epidemiological assessment performed in the current study. It would be prudent to further explore the association of different YE BTs with respect to pre-existing gastrointestinal conditions following yersiniosis notification but using a larger sample size and longer time frame than previously used in New Zealand [19]. Previous studies have reported that YE BT 1A-associated diarrhea may persist for several weeks or months, sometimes involving cyclical periods of acute illness and remission [2,23,89], while other studies have indicated that YE BT 1A is similar to other YE in terms of clinical manifestations and severity [86,90]. *Yersinia* has also been implicated in the causation of inflammatory bowel disease, including Crohn’s disease [91], but not specifically explored for YE BT 1A. A longitudinal study on cases testing positive for YE may elucidate whether YE BT 1A is associated with long-term carriage and opportunistic infections. Findings from the New Zealand case–control study showed that genomic clustering of YE BT 1A clinical cases is uncommon, supporting sporadic infection. Further investigations are also required to refine foodborne risk factors associated with YE BT 1A, which would include comprehensive testing of foods for YE BT 1A.

The database of WGS information can continue to be updated with epidemiological information as it arises; however, this is reliant on isolate referral to the ERL for typing. From 2017, New Zealand diagnostic laboratories have progressively introduced CIDT that includes *Yersinia* (including detection of YE BT 1A) for screening of fecal samples in order to improve diagnosis and enable organism-specific intervention [27]. Diagnostic laboratories are requested to reflex culture all *Yersinia* CIDT-positive samples and forward isolates to the ERL for typing. In the past, laboratories were compliant, with typically >90% of cases being referred [27]. However, the number of isolates forwarded for confirmation and typing, as a percentage of all notifications, has decreased since the adoption of CIDT, from 92% in 2014 to 76% in 2021 [92], and successful detection and identification of *Yersinia* spp. is influenced by the methods used by the laboratories. Failures include the inability to isolate *Yersinia* from the sample, and degraded viability during transit to the ERL and/or insufficient resources to perform the culturing. The latter was apparent during the COVID-19 (2020) pandemic, when laboratory resources were reprioritized. While CIDT may improve the timeliness of pathogen detection, the absence of an isolate for subsequent typing can negatively impact public health research and outbreak detection [27].

As of 2024, all clinical diagnostic laboratories screen stool samples for YE, but CIDT platforms used can differ between laboratories [43]. A previous analysis of New Zealand notification data suggested that the change to methodology had no impact on reporting rates of yersiniosis [92], and the surveillance data analyzed in this study supports this observation for YE BT 1A. However, CIDT has had a negative impact on YP case recognition, as some CIDT panels used (EntericBio^®^Dx, BDMax™, Biofire^®^FilmArray, and Qiastat) do not detect YP. This continues to be of concern as it affects the ability of New Zealand to readily detect YP outbreaks, such as that seen in 2014 [93]. In addition, laboratories may also use sample management criteria that prioritize stool testing for young children, older adults, or other risk groups experiencing gastroenteritis, which may partly explain why these age groups are over-represented in the yersiniosis notifications. Inequities and barriers to accessing primary healthcare and cultural sensitivities preventing the supply of a stool sample by Māori experiencing gastroenteritis may also explain the under-reporting of yersinosis for Māori [94] and will require further research attention.

In addition, in most cases, Cefsulodin-Irgansan-Novobiocin (CIN) Agar, a medium designed for *Yersinia* spp., is used for the isolation of YE, including BT 1A, but it has been previously observed to be of limited use for the differentiation of *Yersinia* spp. [95]. In routine diagnostic workflows, only one or a small number of presumptive *Yersinia* colonies are typically selected from CIN agar for further characterization, which may introduce selection bias and result in the incidental recovery of YE BT 1A, irrespective of the presence of other YE BTs (2/3 or 4). However, if YE BT 1A were a common commensal of the human gastrointestinal tract, it would be expected to be detected frequently and consistently across demographics using CIDT. Information on dual infection(s) detected alongside *Yersinia* spp. for notified cases was a limitation for the epidemiological assessment performed in this study. However, in the New Zealand case–control study, dual infections involving YE BT 1A were rare, with only 0.03% (8/291) of cases identified, suggesting that YE BT 1A may not be widely present as commensal of the gut microbiota [18]. In contrast, the vast majority of YE BT 1A cases within the case–control were not dual infections and showed vast genomic diversity amongst isolates, further supporting the observation of sporadic infection. Furthermore, CIDT panels are limited in their ability to detect pathogens not explicitly targeted by the assay, raising the possibility that other co-infecting undetected pathogens may be contributing to gastrointestinal illness in cases where YE BT 1A is identified. Further investigations may include testing YE-positive fecal samples for different YE and/or other potential pathogens not included in the CIDT panels. Aligning these assessments with enhanced epidemiological data including pre-existing gastrointestinal conditions or symptoms may also be helpful in confirming the role of YE BT 1A in human infection. This, in turn, could help identify YE BT 1A strains associated with infections and genetic factors that allow them to cause infection.

## 5. Conclusions

This study integrated epidemiological, genomic, and in vitro evidence to clarify differences in the distribution and pathogenic potential of YE BTs in New Zealand. Distinct epidemiological trends in age, ethnicity, and regional patterns across YE BTs, particularly for YE BT 1A and YE BT 2/3 and 4, were confirmed. Data to date indicate that the majority of YE BT 1A cases are sporadic infections predominantly occurring in adults, while YE BT 2/3 cases are more frequently notified in young children of Asian ethnicity. Although the four selected YE BT 1A isolates were observed to lack many of the recognized virulence determinants associated with yersiniosis infection and showed limited invasive capacity in 2D cell assays, a preliminary finding from the 3D organ-on-a-chip indicated the capacity of a selected YE BT 1A isolate to elicit an inflammatory IL-8 response, which was an unexpected result. Further studies with additional YE BT 1A and other YE isolates in the 3D organ-on-a-chip model are needed to fully characterize potential pathogenic and cellular mechanisms. Genomic differences, particularly in enterotoxin- and adhesion-related genes, along with methodological limitations in diagnostics and isolate recovery, underscore the complexity of attributing disease causation for YE BT 1A. Collectively, these findings highlight the need to distinguish cases infected with YE BT 1A from those infected with other YE BTs. This practical step will aid public health investigations of cases, while further work is required to collect and analyze enhanced epidemiological data, isolation methodology, and further functional studies to better define the public health significance and infection mechanisms of YE BT 1A.

## Figures and Tables

**Figure 1:**
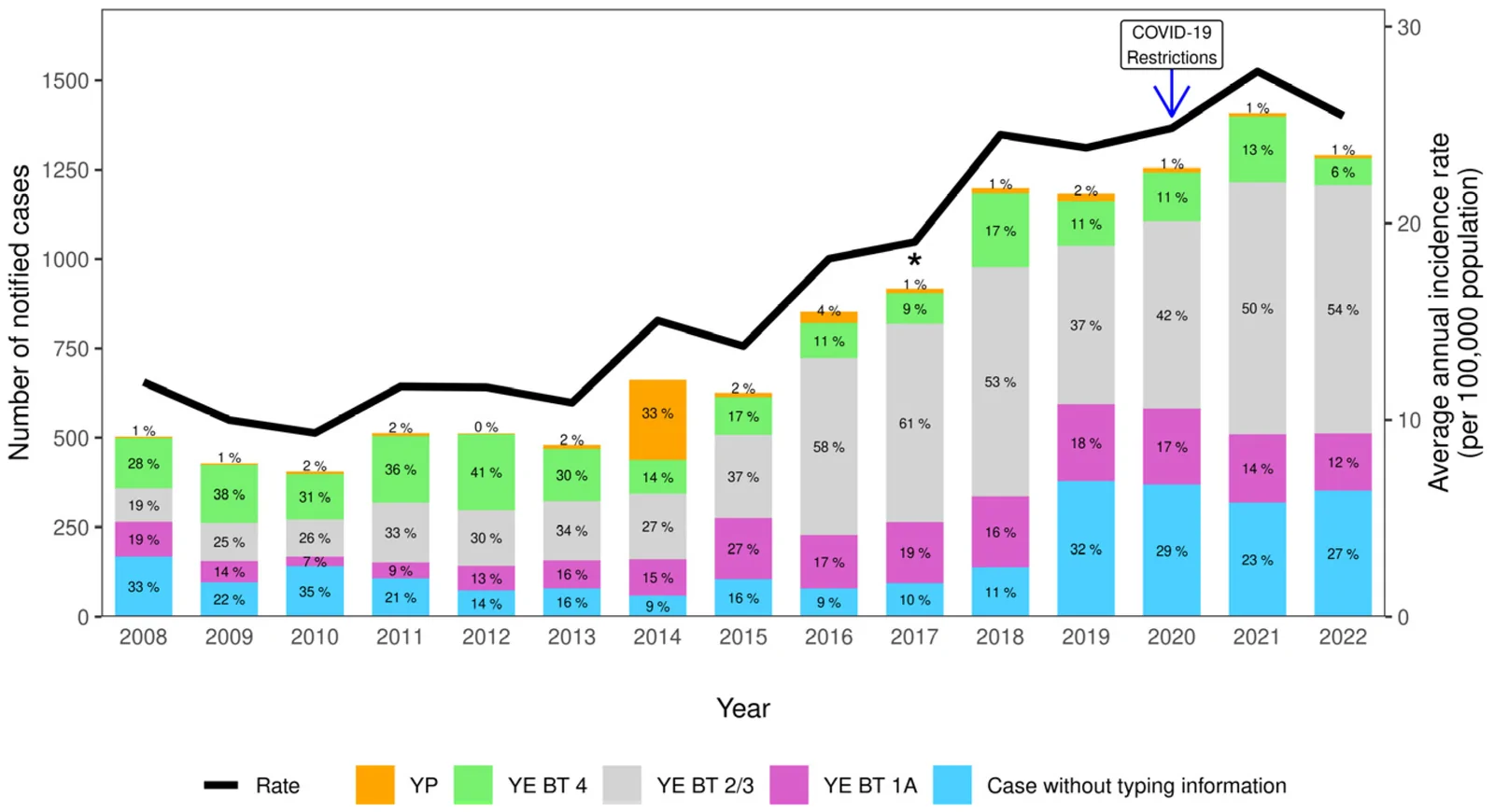
Total number of notified yersiniosis cases in New Zealand between 2008 and 2022 as recorded in EpiSurv. Distribution of cases per annum (and percentage per year) represented according to *Yersinia enterocolitica* (YE) Biotype (BT) (1A, 2/3, and 4) or *Yersinia pseudotuberculosis* (YP). Cases without BT are predominately those where an isolate was not available from the sample, or a BT was not able to be identified. The annual incidence rate was calculated as the number of cases per 100,000 population, based on the Statistics New Zealand mid-year population estimates. * Indicates when the first major diagnostic laboratory introduced a culture-independent diagnostic test that included YE (June 2017). COVID-19 pandemic and the implementation of association national protection measures occurred late March–May 2020 [40].

**Figure 2:**
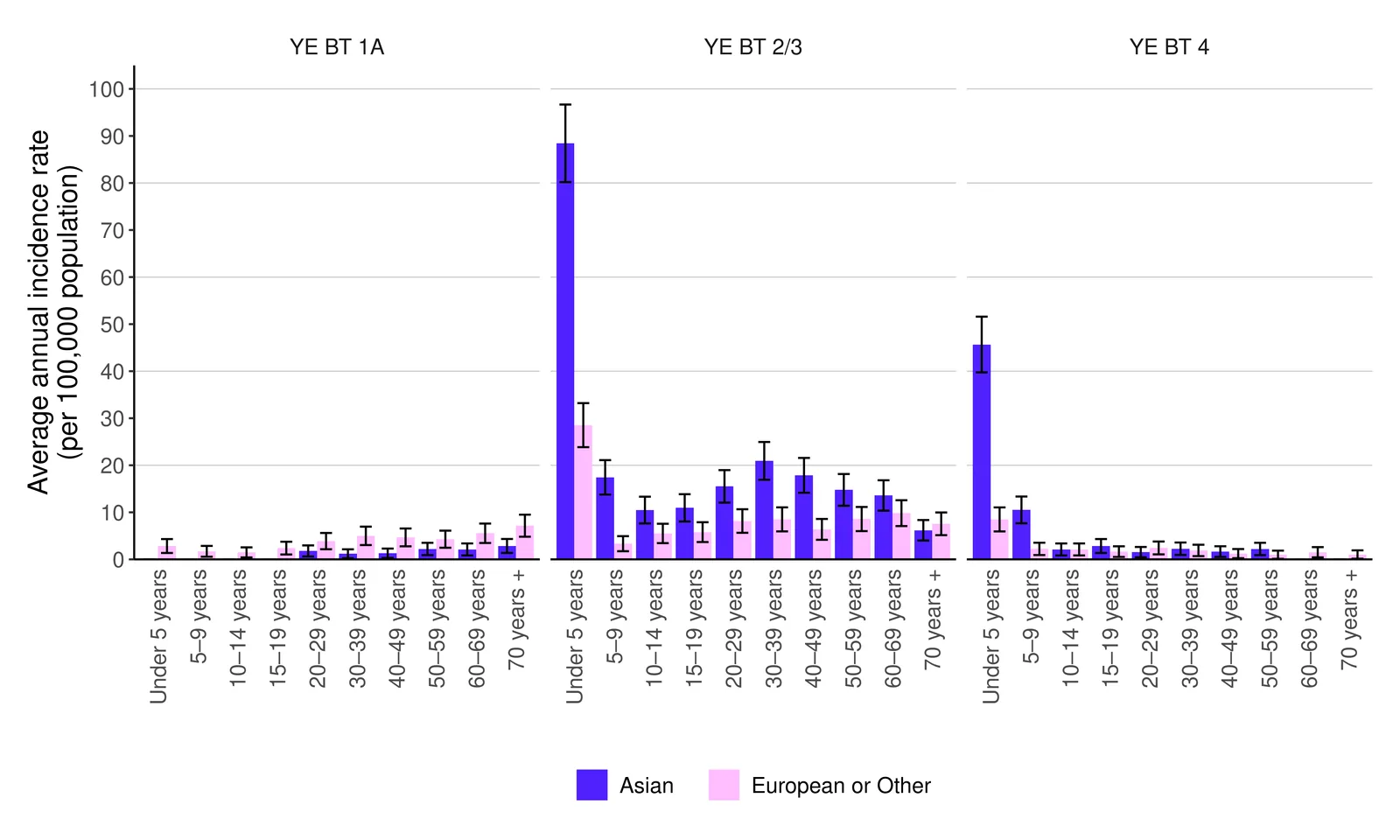
Average annual incidence rates (per 100,000 population) of *Yersinia enterocolitica* (YE) for Biotype (BT) 1A, 2/3, and 4 in New Zealand, by age-group and cases identifying as Asian or European or Other only, 2018–2022. The average annual incidence rate was calculated as the number of cases per 100,000 population, based on the Statistics New Zealand mid-year population estimates. Error bars are 95% confidence intervals. Where the reported case count for a category was fewer than five for 2018–2022, no rate is reported.

**Figure 3:**
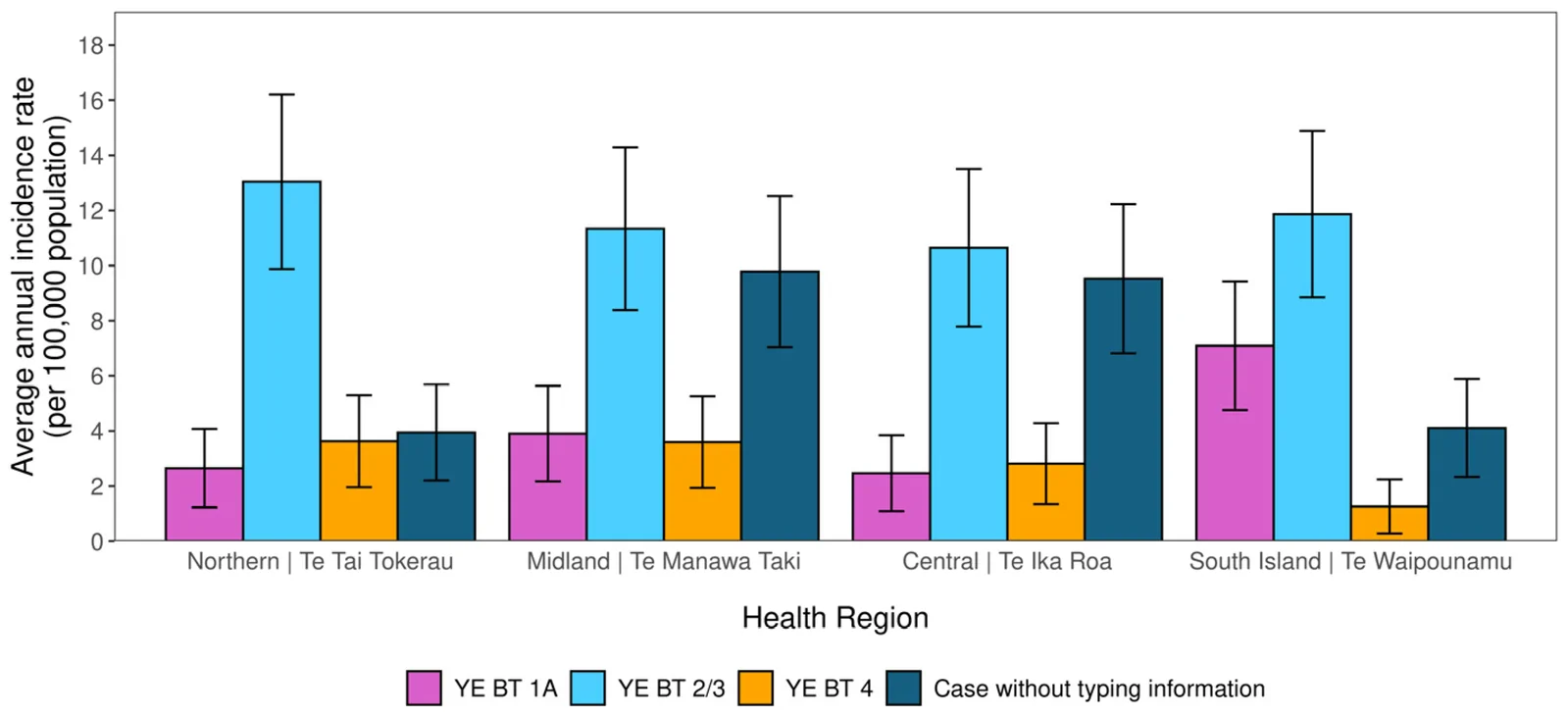
Average annual incidence rates (per 100,000 population) of *Yersinia enterocolitica* (YE) for Biotype (BT) 1A, 2/3, and 4 and for cases where typing information was not available for each health region in New Zealand (2018–2022). Rate calculated as the number of cases per 100,000 population, based on the Statistics New Zealand mid-year population estimates. Error bars are 95% confidence intervals.

**Figure 4:**
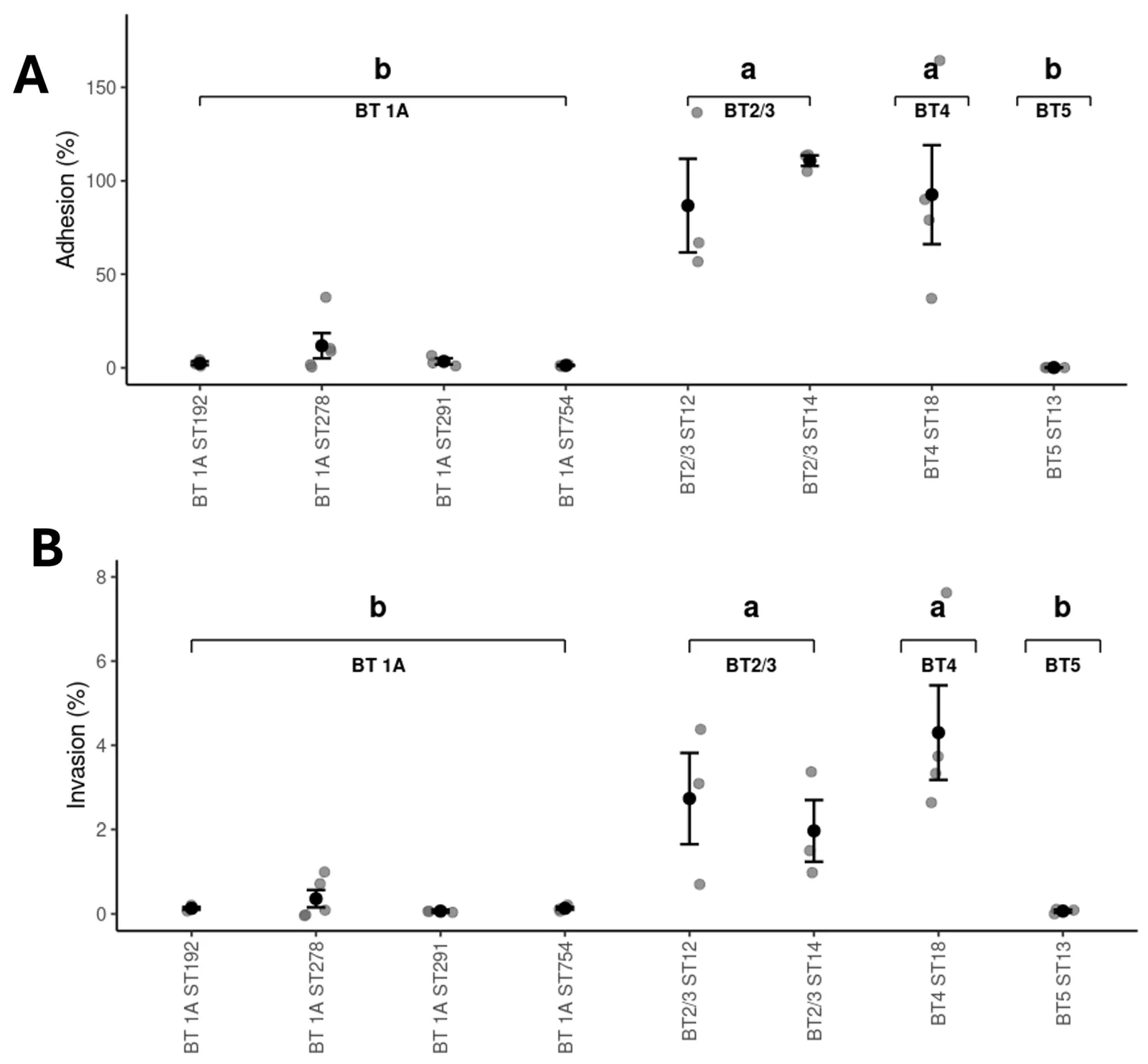
Percent adhesion (**A**) and invasion (**B**) of *Yersinia enterocolitica* (YE) isolates representing different biotype (BT) and multi-locus sequence types (ST) in in vitro Caco-2 cell assays. Gray points represent individual experimental replicates, and black points represent the mean ± standard error. Biotype groupings are indicated by brackets. Different letters indicate significant differences among biotypes as determined by Dunn’s post hoc test with Holm-adjusted *p*-values following a significant Kruskal–Wallis test. Biotypes sharing the same letter were not significantly different (*p* > 0.05).

**Figure 5:**
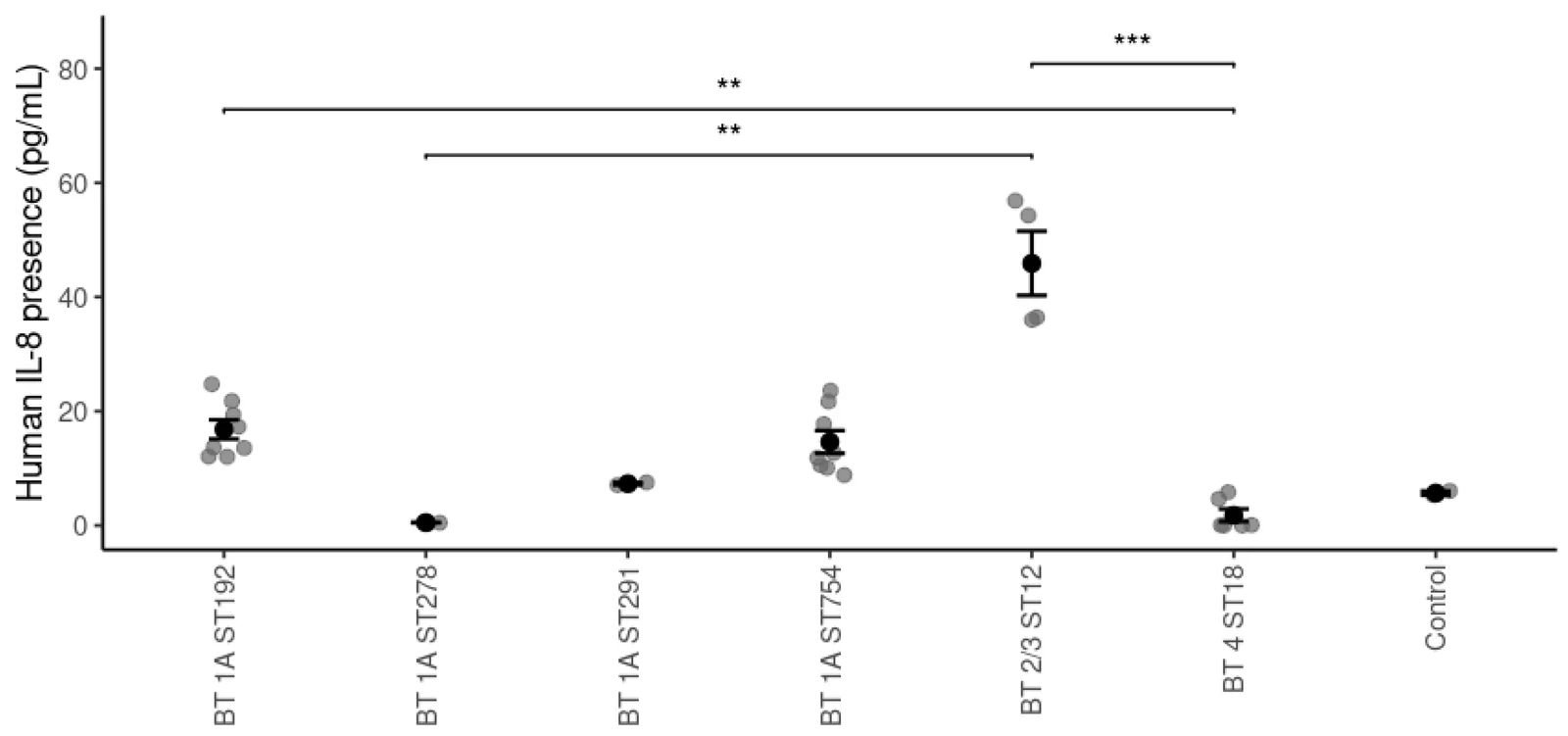
Interleukin-8 (IL-8, pg/mL) detected in cell-free supernatants obtained following the 2D cell assay after the adhesion period (2 h) for *Yersinia enterocolitica* (YE) isolates representing different biotypes (BTs) and multi-locus sequence types (STs). Cell-free supernatants were obtained for 16ER3959, BT 2/3 (n = 4); 22ER1765, BT 4 (n = 6); four BT 1A: 22ER3685 (n = 4), 22ER3398 (n = 8), 22ER3870 (n = 8), and 22ER1202 (n = 2); and uninoculated control (n = 2) (after the 2 h incubation used for the adhesion step only). Supernatants were unavailable for isolate YE BT 2/3 ST14 (22ER3657) and YE BT 5 ST13 (18ER1119) from the original cell assays. Gray points represent individual experimental replicates, and black points represent the mean ± standard error. Statistically significant pairwise differences identified (for all isolates but excluding control) using post hoc Dunn test with Holm adjustments; ** adjusted *p* < 0.01 and *** adjusted *p* < 0.001.

**Figure 6:**
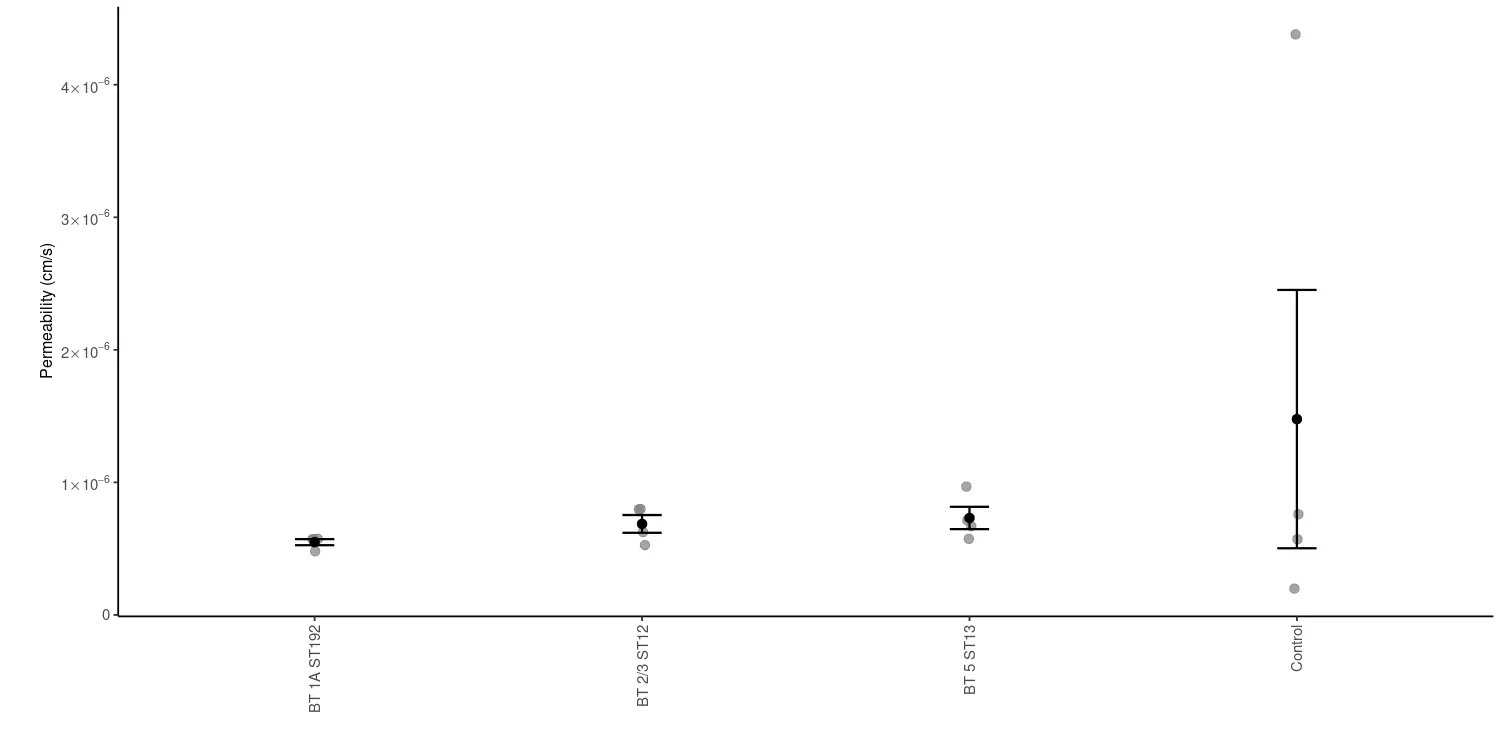
Barrier permeability of Caco-2/HUVEC intestinal organ-chips after 24 h of infection of single isolates of *Yersinia enterocolitica* (YE) Biotype (BT) 2/3, 5, or 1A and multi-locus sequence type (ST) (n = 4 for each isolate). Uninoculated chips were used as a control (n = 4). Gray points represent individual experimental replicates, and black points represent the mean ± standard error.

**Figure 7:**
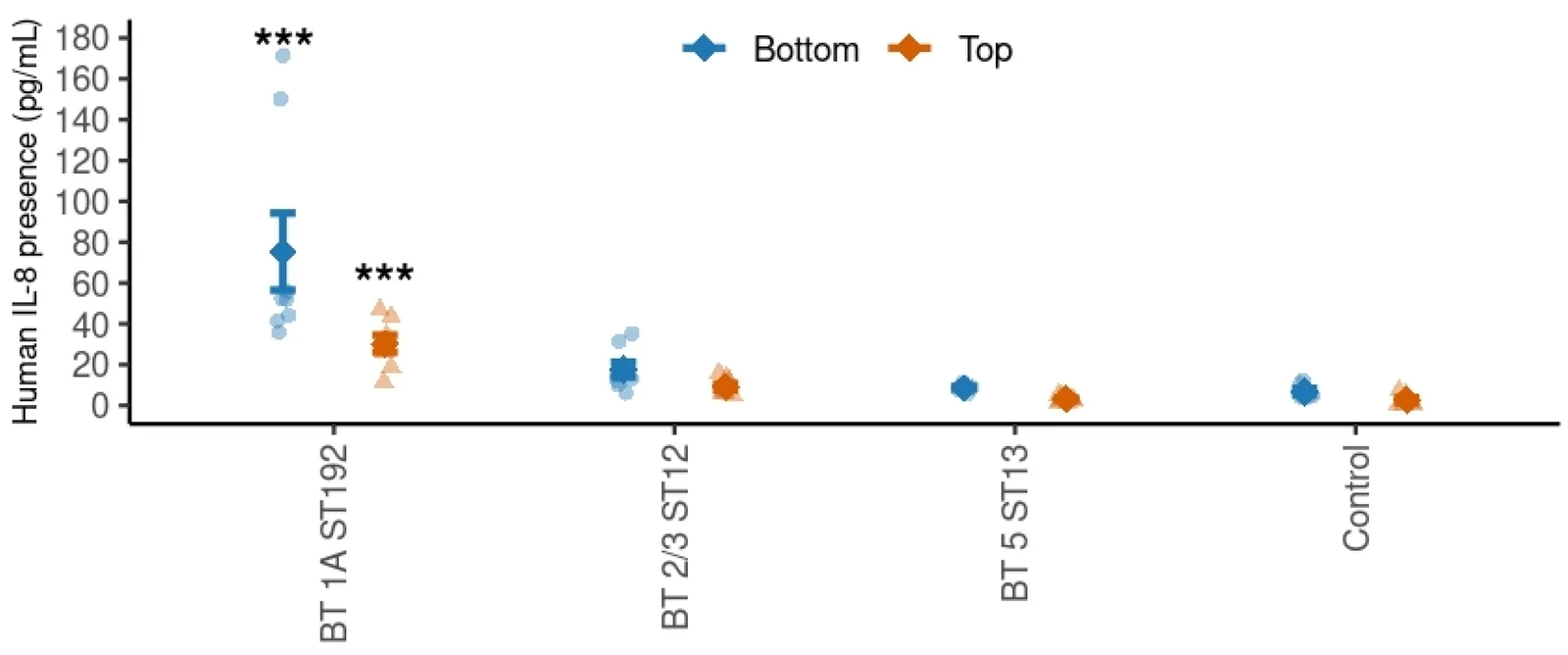
Interleukin-8 (IL-8, pg/mL) detected in cell-free supernatants from the Caco-2 cells (top channel) and HUVEC (bottom channel) within the 3D organ-on-a-chip. *Yersinia enterocolitica* (YE) isolates of Biotype (BT) 2/3, 5, and 1A tested with multi-locus sequence type (ST) noted (n = 8 for each isolate). The control (n = 6) consisted of cell-free supernatant obtained from uninoculated chips. Light blue and orange points represent individual replicates, and darker blue and orange points represent the mean ± standard error. Statistically significant pairwise differences identified for IL-8 concentrations obtained for YE BT 1A ST192 for both the Caco-2 (top) (adjusted *p* = 0.0003) and the HUVEC (bottom) channel compared with the uninoculated control, using post hoc Dunn test with Holm adjustments; *** adjusted *p* < 0.001. No significant differences (*p* > 0.05) were observed for YE BT 2/3 ST12 or YE BT 5 ST 13 for either channel compared with the controls.

**Table 1:** Date of onset of illness reported and hospitalization status for yersiniosis cases 2018–2022 ^a^.

*Yersinia* Species and Biotype (BT)	Yes (%)	No (%)	Total	≥30 Days (%) ^b^	Median (Range) Days	Hospitalization ^c^
Cases with no typing ^d^	847 (54%)	717 (46%)	1564	160 (19%)	11 (1–520)	165/1132 (15%)
YE BT 1A	451 (46%)	526 (54%)	977	94 (21%)	13 (1–641)	88/703 (13%)
YE BT 2/3	1413 (47%)	1595 (53%)	3008	89 (6%)	11 (1–279)	260/1760 (15%)
YE BT 4	308 (42%)	420 (58%)	728	9 (3%)	11 (1–136)	49/377 (13%)
YP	50 (73%)	18 (26%)	68	5 (10%)	12 (1–159)	21/64 (33%)
Total (%) ^e^	3069 (48%)	3276 (52%)	6345	357 (12%)	-	4036/6345 (64%)

^a^ *Yersinia enterocolitica* (YE) Biotypes (1A, 2/3, and 4) and *Yersinia pseudotuberculosis* (YP) cases that had a date of onset of illness recorded (“yes”) and those without (“no”). Excluded: one case of YE BT 1B. Between 4 and 6 cases for each category type had the same date recorded for onset date and notification date, which is not possible. Therefore, these cases were re-assigned as “no” for date of onset of illness reported. Percentage calculated within a *Yersinia* species and BT/no typing. ^b^ Cases with an onset date of illness and notification date ≥30 days. Percentage calculated according to the number of cases that reported ‘yes’ for the same category. ^c^ Number of cases indicating ‘yes’ for hospitalization within a category/number of cases within category with hospitalization status recorded with percentage in brackets. ^d^ Yersiniosis case isolates where no biotype information was available. ^e^ Total ‘yes’ and ‘no’ percentage calculated according to total number of cases (n = 6345).

**Table 2:** Key virulence-associated genes that differed between *Y. enterocolitica* biotypes screened ^a^.

Gene	YE BT 1A			YE BT 2/3			YE BT 4			YE BT 5
	Clinical (n = 59)	Food (n = 227)	Total (n = 286)	Clinical (n = 101)	Food (n = 66)	Total (n = 167)	Clinical (n = 8)	Food (n = 7)	Total (n = 15)	Total (n = 2) ^c^
*ail* ^a^	0	0	0	101 (100%)	66 (100%)	167 (100%)	8 (100%)	7 (100%)	15 (100%)	2
*invA*	59 (100%)	224 (99%)	283 (99%)	101 (100%)	66 (100%)	167 (100%)	8 (100%)	7 (100%)	15 (100%)	2
*invB*	48 (81%)	187 (82%)	235 (82%)	0	0	0	0	0	0	0 ^d^
TTSS ^b^	0	0	0	101 (100%)	65 (98%)	166 (99%)	8 (100%)	7 (100%)	15 (100%)	2
*cpsG*	24 (41%)	66 (26%)	90 (31%)	0	0	0	0	0	0	0
*galE*	19 (32%)	42 (19%)	61 (22%)	0	0	0	0	0	0	0
*gmd*	19 (32%)	42 (19%)	(61 (22%)	0	0	0	0	0	0	0
*rfbF*	26 (44%)	57 (25%)	83 (29%)	0	0	0	0	0	0	0
*rfbG*	16 (27%)	35 (15%)	51 (18%)	0	0	0	0	0	0	0
*yadA*	0	0	0	101 (100%)	65 (98%)	166 (99%)	3 (38%)	1 (14%)	4 (27%)	2
*yaxA*	59 (100%)	227 (100%)	286 (100%)	101 (100%)	66 (100%)	167 (100%)	8 (100%)	7 (100%)	15 (100%)	0
*yaxB*	59 (100%)	227 (100%)	286 (100%)	101 (100%)	66 (100%)	167 (100%)	8 (100%)	7 (100%)	15 (100%)	2^e^
*ystA*	0	0	0	101 (100%)	66 (100%)	167 (100%)	8 (100%)	7 (100%)	15 (100%)	2
*ystB*	59 (100%)	227 (100%)	286 (100%)	0	0	0	0	0	0	0
*yscP*	0	0	0	101 (100%)	65 (98%)	166 (99%)	8 (100%)	7 (100%)	15 (100%)	0

^a^ As inferred using whole-genome sequencing data from previous studies [18,27] and using ABRIcate and the Virulence Factor Database [31,32,33], using a minimum DNA percentage identity and coverage of 80% and 60%, respectively. The *ail* gene was negative for all YE genomes using these tools. Annotation of all genomes confirmed the presence of the *ail* gene for all YE BT 2/3, 4, and 5 isolates, but the BT 5 isolates also had a 27 base-pair insertion. All YE BT 1A were negative for *ail.* ^b^ Genes associated with the Type III secretion system (TTSS) [42]. ^c^ YE biotype 5 isolates were obtained from sick animals [27]. ^d^ Detection of <70% coverage of the gene. *yaxB* had 76% coverage of the gene.

## Data Availability

Raw sequence reads for all isolates analyzed in this study are available at the National Centre for Biotechnology Information (NCBI) short read archive (SRA) with BioProject number PRJNA 1142067.

## Abbreviations

The following abbreviations are used in this manuscript:

2D/3D	Two- or three-dimensional
ANOVA	Analysis of variance
BT/BTs	Biotype/biotypes
CIDT	Culture-independent diagnostic test
CFU	Colony forming units
COVID-19	Coronavirus-disease of 2019
DMEM	Dulbecco’s Modified Eagle Medium
ECM	Extra cellular matrix
ELISA	Enzyme-linked immunosorbent assays
ERL	Enteric Reference Laboratory
HUVEC	Human umbilical vein endothelial cells
IL	Interleukin
NCBI	National Centre for Biotechnology Information
MELAA	Middle Eastern/Latin American/African
MOI	Multiplicity of infection
OD	Optical density
PBS	Phosphate-buffered saline
PHF	New Zealand Institute for Public Health and Forensic Science
pYV	Plasmid of Yersinia virulence
qPCR	Quantitative polymerase chain reaction
SRA	Short read archive
ST/STs	Multi-locus sequence type(s)
TE	Tris-EDTA
TNF-α	Tumor necrosis factor-alpha
TSA	Tryptone soya agar
TSB	Tryptic soya broth
TTSS	Type III secretion system
USA	United States of America
UV	Ultraviolet
VFDB	Virulence factor database
WGS	Whole-genome sequencing
YE	*Yersinia enterocolitica*
YP	*Yersinia pseudotuberculosis*
